# The Book World of Medicine and Science

**Published:** 1901-12-21

**Authors:** 


### The Book World of Medicine and Science.

A Retrospect of Surgery during the Past Century, being the Hunterian Oration of the Hunterian Society, 1901. By John Poland, F.R.C.S., Surgeon to the City Orthopædic Hospital, Senior Surgeon to the Miller Hospital. (London: Smith, Elder and Co. 1901. Pp 97. Price 5s.)

This is an extremely interesting and well-arranged account of the enormous progress made in the science and art of surgery since Wiseman established it as a distinct branch of medicine about the middle of the seventeenth century, but most will agree that Hunter, who died in 1793, was the real founder of scientific surgery. The enormous influence which the study of pathology has had upon the principles of surgical procedure and the great effects of anæsthetics and antiseptics upon operations are clearly demonstrated, but the lecturer calls attention to the present high mortality from anæsthetics, and hopes that the new century will be productive of better teaching and better apparatus in connection with that branch of practice. Mr. Poland thinks the modern surgeons do not equal John Hunter in manual dexterity, and recommends that "all should avail themselves in early youth of the opportunities of learning to use a saw and handle a chisel and hammer in the carpenter's shop." A large part of the work deals with the diagnosis and treatment of fractures, and points out the great advantages of the present-day conservative methods of surgery over the old practice of immediate amputation. Timely warning is given to those who are apt to use or abuse skiagraphs as a means of diagnosis, for it is becoming more evident that a great deal of experience is necessary before the exact condition can be interpreted in all cases from the photographic plate. The treatment of lupus by the X rays and the use of electricity in the diagnosis and treatment of paralysis are also lightly touched upon at the end of the address.

On the Cure of the Morphia Habit. By Oscar Jennings, M.D. Second edition. (Baillière, Tindall and Cox. 1901. 3s. 6d. net.

This volume is worth perusal and should be read by every practitioner in charge of a morphia subject. If the language is at times self-laudatory, it is a record of one who has paid attention to the subject, who has had opportunities for observation, and has been (he owns frankly) a victim himself, and thereby understands that difficult psychological study—the deranged intellect of the morphia habit. The brochure is avowedly addressed as much to patients as physicians, the author claiming that the evasive, argumentative mental state of morphia subjects, mostly of superior intelligence and social status, demands a simple statement of the difficulties of their cure for their own study. Dr. Jennings refuses to believe that *habitués* can be cured by brute force, by withdrawing the drug suddenly under prison supervision. If such cases can be thus totally deprived in apparent safety they always relapse. He strongly insists on the effect of continued morphia poisoning on the heart and vaso-motor system, and the necessity for the substitution of some cardiac stimulant; and he devotes considerable space to the relative advantages of such drugs as sparteine, trinitrine, and digitalis. Great importance is attached to the use of sphygmographic investigation of cardiac and vascular conditions, which cannot be adequately gauged by the finger on the pulse. The hyperacidity of the stomach, which is a feature of the craving stage, must be met; the gastric pain and discomfort which induces patients to indulge in irregular and inordinate meals, must be controlled by proper and regular feeding. Value is attached to the use of hot-air baths, and systematic gentle exercise, such as bicycling. In "tailing off" the doses of morphia the final administration of minimum doses (under 2 grains) should, for various reasons be by rectal injection.

A Practical Guide to the Administration of Anæsthetics. By R. J. Probyn-Williams, M.D. (London: Longmans, Green and Co. 1901. Price 4s. 6d. net.)

In writing this book the author has endeavoured, he says, to supply essential points in as small a form as possible, so as to meet the wants of students. On the whole the book will serve its purpose as a guide to the administration of anæsthetics. The various substances employed and the methods by which they are to be administered are carefully and minutely described, and great pains are taken in explaining the sometimes rather complicated internal arrangements of the several forms of apparatus which are in use for the purpose. For the administration of ether Clover's smaller inhaler is described as the standard apparatus, although in some cases, as when ether follows full anæsthetisation by gas, Ormsby's apparatus is shown to have certain advantages from the larger air-ways and the possibility which it offers of giving a great strength of vapour. For the administration of chloroform the author says that the best form of inhaler is a special form of Skinner's mask, a figure of which he gives. But he also speaks favourably of Dudley Buxton's modified Junker's, which is curious after what he says concerning the evils of confining the vapour. Still more curious is it that he should speak slightingly of an inhaler which he refrains from naming, but indicates by describing it as having "a feather at the apex" of the face piece. These face pieces, he says, "are a source of danger, as the feather moves with such a minute puff that the anæsthetist may be lulled into a sense of false security by seeing some movement of the feather, when in reality very little respiration is taking place." In saying this, Dr. Probyn-Williams evidently fails to recognise what is the real object and advantage of the feather. His remarks about chloroform form, indeed, the weak spot on the book. The statement in page 146 that in the air from the chloroform bottle of a Junker's inhaler "the strength of the chloroform cannot pass above 4 per cent.," must, we think, be accepted with considerable caution, as also the statement that in the Dudley Buxton inhaler "there is no fear that the vapour will become too strong—that is, that it will contain more than 4 per cent. of chloroform." This, of course, depends on how the mask fits, on how long the intervals are between the inspiration, and how vigorously the anæsthetist works the pump. In describing the Junker inhaler he entirely misses the point; the advantage of this apparatus, as we take it, being not that it is impossible to give too strong a vapour, for this is very possible, but that it is impossible for any vapour at all to reach the patient except by the conscious act of the anæsthetist, who is thus enabled (by aid of the "feather" if his apparatus has one, or if not by some other more rough-and-ready method) to time the administration in proper relation to the respiration and so to regulate with some degree of precision the quantity inspired. Of this he says nothing. The directions given for the administration of the other anæsthetics are, however, satisfactory.

